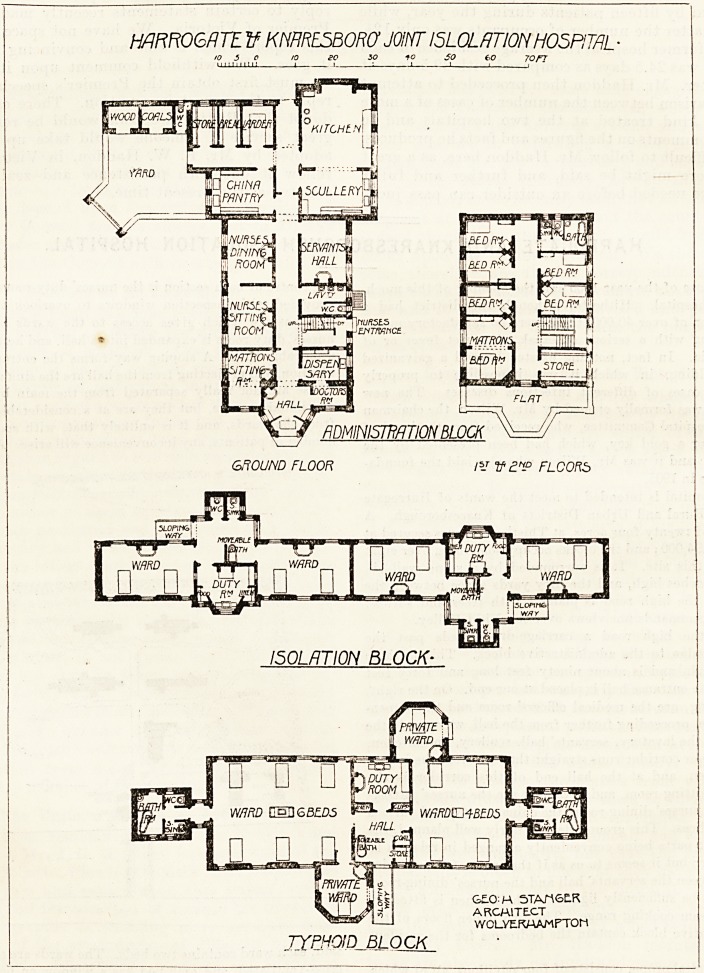# Harrogate and Knaresborough Isolation Hospital

**Published:** 1906-06-30

**Authors:** 


### HARROGATE AND KNARESBOROUGH ISOLATION HOSPITAL.

The close of the year 1905 saw the opening of this much-needed hospital. Hitherto, although the district had a population of over 40,000, there were no satisfactory means of dealing with a serious outbreak of scarlet fever or of diphtheria. In fact, nothing existed beyond a galvanized iron building, in which it was impossible to properly separate cases of different infectious diseases. The new building was formally opened by Mr. Wilson, the chairman of the Hospital Committee, who received from the hand of the Mayor a gold key, which had been presented by the architect; and it was Mr. Wilson who also laid the foundation-stone in 1903.

The hospital is intended to meet the wants of Harrogate and the Rural and Urban Districts of Knaresborough. A fine site of twenty-four acres, at Thistle Hill, was secured at a cost of £4,000; and the blocks occupy something over eight acres of this site. It is surrounded by a stone wall six feet six inches high, and the fifty yards space between the wall and the high road is planted with trees and shrubs. The site commands fine views over the Nidd Valley.

From the high road a carriage-drive leads past the porter's lodge to the administrative block. This is three stories high, and is about ninety feet long and forty feet wide. The entrance hall is placed at one end. On the right, on entering, are the medical officers' room and the dispensary; then, proceeding further from the hall, we come to the staircase, the lavatory, servants' hall, scullery, and kitchen. A passage or corridor runs straight through from the hall to the kitchen, and at the hall end of this corridor is the matron's sitting-room, and beyond it are the nurses' sitting-room, the nurses' dining-room, the china pantry, and various kitchen offices. This ground floor is fairly well planned, the component parts being conveniently arranged in relation to each other; but it seems to us as if that part of the corridor lying between the servants' hall and the nurses' dining-room might not be sufficiently lighted. The kitchen is fitted up with a steam cooking range. The two upper floors of the administrative block contain the bedrooms for the resident staff.

The hospital proper consists of four pavilions, all of which are placed within easy distance of the administrative block. One of these pavilions is for scarlet fever, one for diphtheria, one for typhoid fever, and one is for the isolation of doubtful cases. We give herewith the plan of the latter, and also that of the typhoid pavilion.

The isolation block is a long, narrow pavilion, divided into two equal sections presumably for the two sexes, and each section is divided into two wards having two beds each. In the centre of each section is the nurses' duty-room, which is provided with inspection windows to overlook the wards. The passage which gives access to the wards and to the nurses' duty-room is expanded into a hall, and here is placed a movable bath. A sloping way forms the entrance to the pavilion, and projecting from the hall are the sink and closet. These are not really separated from the main by a cross-ventilated passage, but they are at a considerable distance from the wards, and it is unlikely that, with such a small number of patients, any inconvenience will arise. As already said, each ward contains two beds. The wards are efficiently cross-ventilated, and each bed has a window on both sides. Another window at the end would have made the ward look more cheerful, but there is plenty of light as it is.

The typhoid fever block contains one ward for four beds and one ward for six beds, and also two single-bedded wards. There is nothing special to remark about this pavilion. It is an ordinary plan. The wards seem well lighted, and, except in one case which it would have been impossible to avoid, every bed in the wards has a window on both sides, and the sanitary annexes are cut off from the ward by properly cross-ventilated passages. Here also the free ends of the wards are destitute of windows, and there are no verandahs. It was not considered necessary to provide a pavilion for small-pox, as this disease will be treated in the old hospital at Killinghall Moor.

The internal arrangements of the various pavilions are similar to each other, and are said to be very good. The ventilation throughout has been carefully seen to. The wards are heated by Shorland's grates, supplemented by steam radiators, and this combination ensures plenty of warmth. Outlets for vitiated air are provided near the ceiling levels, but it is not stated what, if any, method of extraction is relied on. To prevent accumulation of dust the corners of the wards have been rounded off, and the ward floors are laid down with oak blocks. The lighting is by gas on the incandescent principle, and water is obtained from the Harrogate Corporation works. Each block is drained separately, and the drain is intercepted, disconnected, and ventilated before it joins the main drain. Further a flushing tank containing 560 gallons of water is placed at the head of the main drain.

Judging from the plans submitted to us, we think that the hospital is tolerably well adapted for the treatment of infectious diseases. So far, so good; but we can only remark once more that the plans of these isolation hospitals, as a rule, show a great want of originality in conception. The Local Government Board models are too closely followed. Indeed, it is doubtful whether any marked departure from these models would be understood in a right sense or adequately appreciated. This very hospital was built from plans selected in open competition; and it is consistent with our knowledge that, what we should look on as a better plan was sent in and failed to get even a premium, although it could probably have been built for several thousand pounds less than the selected one. According to the *Bradford Observer*, the Harrogate Hospital has cost between £25,000 and £30,000, but most likely this includes the cost of the site and the boundary wall. At £25,000 the cost of the sixty beds would work out at about £400 a bed; and for this sum and number of beds everything should be not only good, but more than good. The *Bradford Observer* adds, what is apparently the opinion of the Chairman of the Finance Committee, that "had the Corporation to set about the business again they would not carry it out on the lines that had been adopted."

The architect was Mr. Stanger, of Wolverhampton, and the contractors were Messrs. Simpson and Son, of Harrogate. Mr. Rudd, of Harrogate, was responsible for the joiners' work; and the plumbing was done by Mr. G. Thompson, of Leeds.

## Figures and Tables

**Figure f1:**
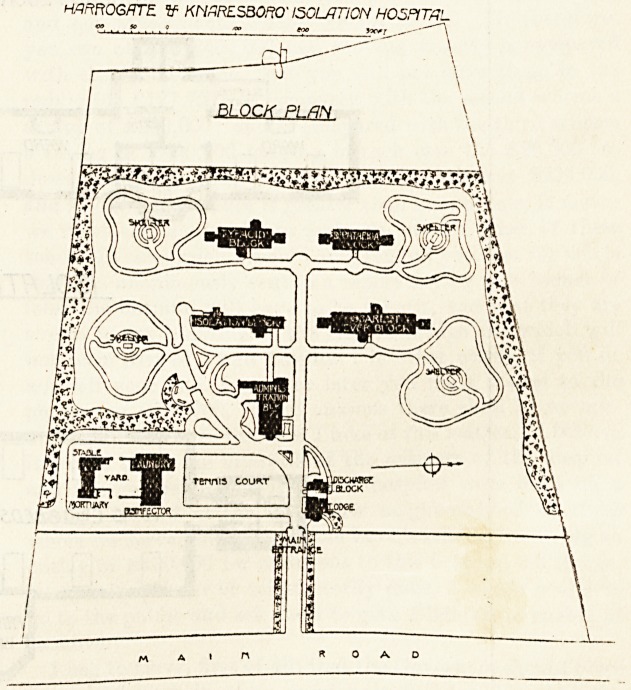


**Figure f2:**